# DNA as a Chiral Scaffold for Asymmetric Synthesi

**DOI:** 10.3390/molecules171112792

**Published:** 2012-10-31

**Authors:** Soyoung Park, Hiroshi Sugiyama

**Affiliations:** 1 Department of Chemistry, Graduate School of Science, Kyoto University, Kitashirakawa-oiwakecho, Sakyo-ku, Kyoto 606-8502, Japan; 2 Institute for Integrated Cell-Material Sciences (iCeMS), Kyoto University, Yoshida-ushinomiyacho, Sakyo-ku, Kyoto 606-8501, Japan; 3 CREST, Japan Science and Technology Corporation (JST), Sanbancho, Chiyoda-ku, Tokyo 102-0075, Japan

**Keywords:** DNA, asymmetric synthesis, helical chirality, hybrid catalyst

## Abstract

The application of DNA-based hybrid catalysts for enantioselective synthesis has recently emerged. These catalysts, self-assembled from DNA and a metal complex with a specific ligand through supramolecular or covalent anchoring strategies, have demonstrated high enantioselectivity in a variety of carbon-carbon or carbon-heteroatom bond-forming reactions and have expanded their role in asymmetric catalysis. In this review, we summarize the advent and significant progress of DNA-based asymmetric catalysis and discuss remaining challenges in using DNA as a chiral scaffold. We hope that this review will inspire many of today’s active scientists in asymmetric catalysis.

## 1. Introduction

Nature selects only one enantiomer of a molecule to function appositely in a living cell and produces chiral molecules using enzymes that catalyze asymmetric reactions with exquisite selectivity beyond conventional synthetic methods. Taking inspiration from this prowess, many researchers have investigated stereoselective hybrid catalysts that combine the catalytic power of a metal complex with the exquisite chirality of a biomolecule as alternative tools for the synthesis of enantiomerically pure compounds [1,2,3,4,5,6]. Considering the broad scope of transformations enabled by metal-catalyzed reactions and the vast diversity of natural and artificial biomolecules, this combination seems to offer a very promising strategy.

Among various biomolecules, the application of DNA-based hybrid catalysts for enantioselective synthesis has emerged only recently [7,8,9]. Although helical chirality has received attention as a simple and highly organized chiral structure for asymmetric synthesis and significant advances have been reported, earlier workers relied on the use of synthetic helical polymers [10,11,12,13,14,15,16,17]. Considering the toil and effort required for the development of helical synthetic polymers, the use of DNA, one of the most plentiful and naturally occurring helical polymers, is a gift from nature to synthetic chemists. DNA is a promising candidate as a source of chirality in asymmetric catalysis in many respects. Compared with RNA and proteins, DNA is chemically more stable and thus it is easy to handle in chemical reactions. DNA is a cheap and readily available biopolymer. For example, 1 kg of bulk DNA can be bought for US$ 100–200, and 1 g of purified salmon testes DNA (st-DNA) for about US$ 77. Another virtue of DNA is its structural properties, which can be readily controlled by laboratory methods. Although mainly B-DNA has been observed in organisms, there are many possible DNA conformations, including the A-DNA and Z-DNA forms. In addition, DNA can form not only a double helix, but also triplex and quadruplex forms. The high specificity of the A–T and G–C Watson–Crick hydrogen-bonding interactions enables the construction of various artificial structures on the basis of the simple four-letter alphabet. Furthermore, DNA is a suitable starting point for the development of water-compatible catalysts because of its high solubility in water. Asymmetric catalysis in water is a very important research area for green chemistry. Herein, we will discuss the advent and the significant progress of DNA-based asymmetric catalysis and highlight the remaining challenges in using DNA as a chiral scaffold. 

## 2. The Advent of DNA Hybrid Catalyst and the Catalyst Design

**Scheme 1:**
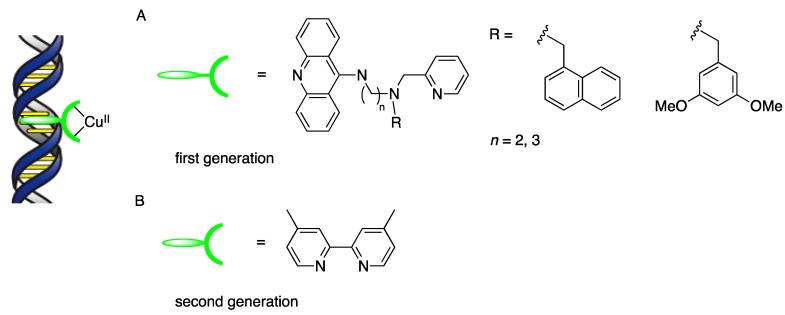
Concept illustration of a DNA-based hybrid catalyst and representative binding ligands.

The application of DNA-based hybrid catalysts to asymmetric catalysis was first reported in the form of a copper(II)-catalyzed Diels–Alder reaction by Feringa and Roelfes in 2005 [18]. They introduced the novel concept of a DNA-based hybrid catalyst based on a supramolecular assembly with three essential components: (1) a catalytically active metal complex; (2) DNA as a chiral scaffold; (3) a binding, nonchiral ligand to connect the metal complex to the DNA. First-generation binding ligands possessed three functional parts in one molecule: a DNA intercalating domain such as 9-aminoacridine, a spacer moiety, and a metal-binding group (Scheme 1A). The enantioselectivity of the Diels–Alder reaction proved to be dependent on the substituent, R, of the ligand, and on the spacer length. Subsequently, it was found that nearly flat, symmetrical bipyridine-type ligands, e.g., 4,4'-dimethyl-2,2'-bipyridine (dmbpy), could be applied successfully to DNA-based asymmetric catalysis (Scheme 1B) [19]. With this simple second-generation ligand, the metal-binding domain and DNA-binding group are incorporated into one moiety; thus, a spacer is no longer required. A DNA-based hybrid catalyst consisting of Cu(dmbpy) and st-DNA [20] has afforded the highest enantioselectivities of reported copper(II)-catalyzed reactions, such as Diels–Alder, Michael addition, and Friedel–Crafts alkylations. On the other hand, in enantioselective *syn* hydration of enones and enantioselective oxa-Michael addition, the first-generation ligands gave higher enantioselectivities than second-generation ligands. We revisit these reactions later in this review.

**Scheme 2:**
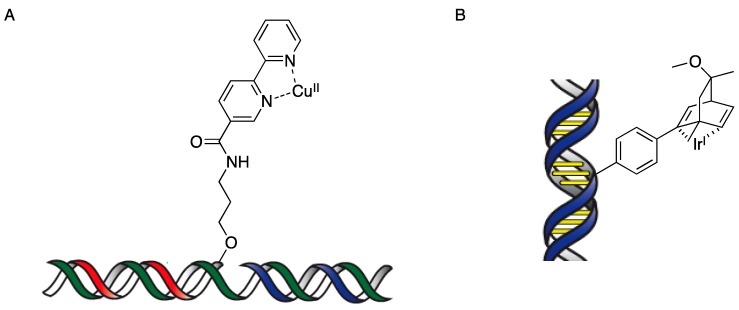
Covalent anchoring strategy for DNA-based asymmetric catalysis. (**A**) bipyridine ligand; (**B**) Diene ligand.

In addition to the supramolecular assembly strategy, a covalent anchoring strategy has been developed. Roelfes and co-workers developed DNA-based hybrid catalysts containing a covalently anchored metal complex and demonstrated their effectiveness in asymmetric Diels–Alder reactions (Scheme 2A) [21]. This approach involves a functionalized oligonucleotide by synthesis of an oligonucleotide with a second-generation ligand, 2,2'-bipyridine, an unfunctionalized oligonucleotide, and a template oligonucleotide strand with a complementary sequence. Through hybridization, the copper(II) complex is positioned in the DNA. Jäschke and co-workers developed a DNA-based hybrid catalyst by introducing a covalent anchored diene ligand for allylic amination by a DNA-diene- iridium(I) hybrid catalyst (Scheme 2B) [22]. Even though the covalent anchoring strategy has the important advantage that it enables the precise positioning of the metal complex in the DNA and fine-tuning with the specific DNA sequence, it requires time-consuming and costly processes compared with the supramolecular assembly strategy in which a DNA-based hybrid catalyst is formed *in situ* by the simple addition of copper(II)-ligand complex to DNA.

## 3. The Reaction Scope of DNA-Based Asymmetric Synthesis

### 3.1. Carbon-Carbon Bond Forming Reaction

**Scheme 3:**
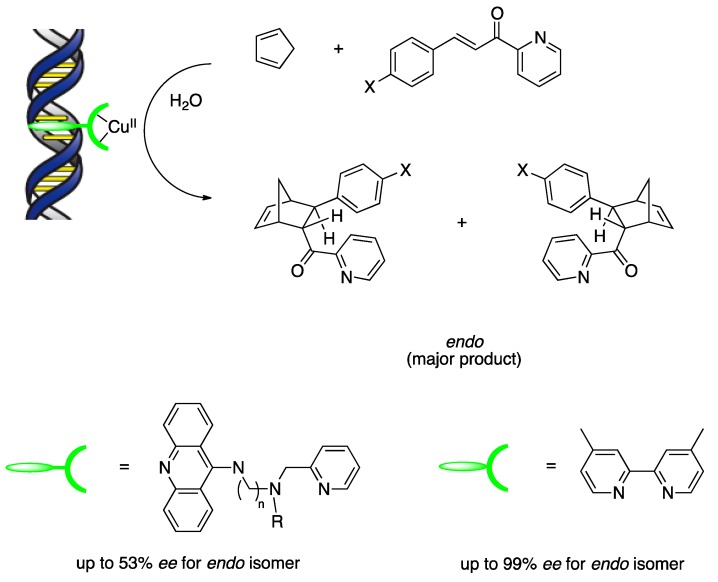
Diels–Alder reaction catalyzed by a supramolecular assembly of copper(II)-ligand complex and DNA.

**Scheme 4:**
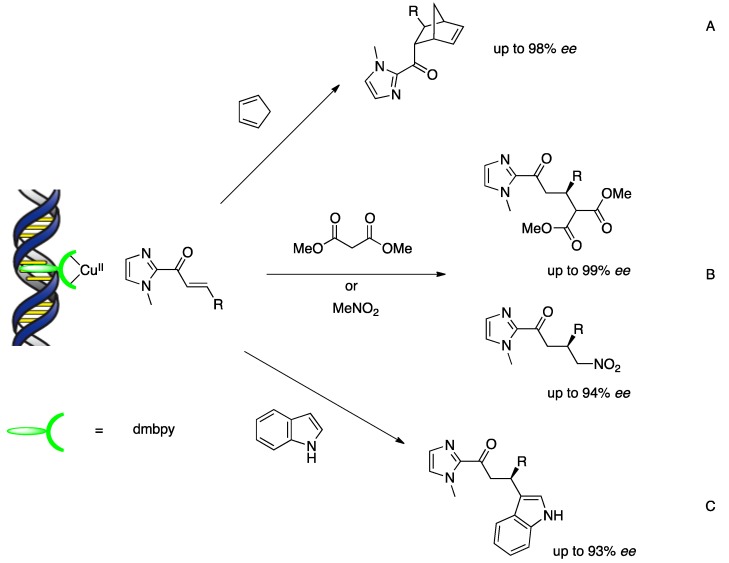
Reaction scope of carbon-carbon bond forming reaction by DNA-based hybrid catalysts. (**A**) Diels–Alder reaction; (**B**) Michael addition; (**C**) Friedel–Crafts alkylation.

The potential of DNA-based hybrid catalysts for asymmetric catalysis was demonstrated for the first time in a copper(II)-catalyzed Diels–Alder reaction between cyclopentadiene and an azachalcone (Scheme 3) [18]. Using DNA hybrid catalysts formed by supramolecular assembly with first-generation ligands, up to 53% ee was obtained for the *endo* isomer. With respect to substrate scope, the dienophiles possessing the 2-acyl pyridine moiety, which can chelate the copper(II) complex, were chosen for both activity and enantioselectivity. This feature is a potential concern for practical applications in synthesis because the pyridyl group cannot be removed or transformed easily. However, the pyridine moiety could be replaced by a readily removable imidazole auxiliary. This alternative α,β-unsaturated 2-acyl imidazole dienophile bound to the copper(II) ions in a bidentate fashion under aqueous conditions and gave Diels–Alder adducts with high enantioselectivities in the presence of the second-generation ligand (up to 98% *ee*) (Scheme 4A) [23]. This improvement has proved useful in a variety of Lewis acid-catalyzed reactions as well as in Diels–Alder reactions. A DNA-based hybrid catalyst self-assembled from st-DNA and a copper complex with dmbpy was used to promote a highly enantioselective Michael reaction in water (Scheme 4B) [24]. The Michael adducts were obtained with up to 99% *ee* when dimethyl malonate was used as the nucleophile and an α,β-unsaturated 2-acyl imidazole was used as the Michael acceptor. Nitromethane was also a good nucleophile in this reaction: the corresponding products were formed with up to 94% *ee*. A DNA-based hybrid catalyst was also applied successfully to the Friedel–Crafts alkylations of indoles and high enantioselectivities up to 93% *ee* were obtained (Scheme 4C) [25].

### 3.2. Carbon-Heteroatom Bond Forming Reaction

**Scheme 5:**
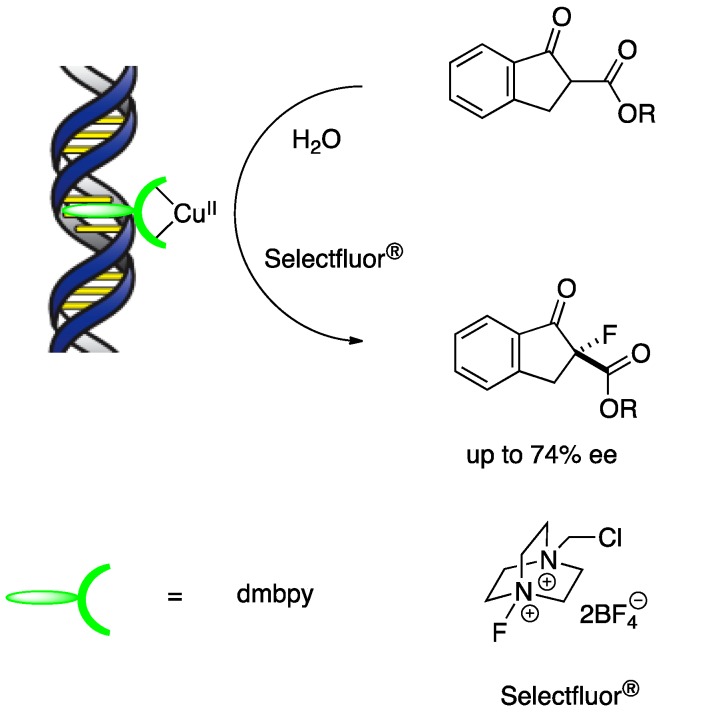
Enantioselective C-F bond formation catalyzed by copper(II)-dmbpy-DNA.

The creation of carbon–heteroatom bonds with a DNA-based hybrid catalyst has also been demonstrated. Shibata, Toru, and co-workers developed the first DNA-based asymmetric C–F bond-forming reaction by combining a chemical fluorination procedure and the DNA hybrid catalyst system (Scheme 5) [26]. 

**Scheme 6:**
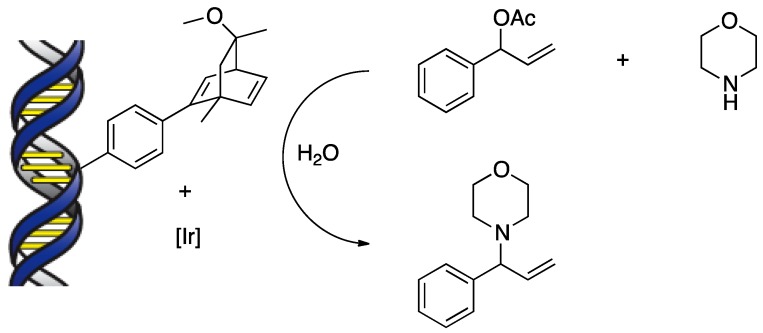
Allylic amination by an iridium(I)-diene-DNA hybrid catalyst.

The fluorination of indanone β-ketoesters with a copper(II)-dmbpy-DNA catalyst was carried out in an aqueous buffer with Selectfluor as the fluorine-transfer reagent. In the presence of dmbpy, fluorinated products were obtained with good DNA-induced enantioselectivity (up to 74% *ee*). Jäschke and co-workers demonstrated that a DNA-based system could be utilized for an allylic substitution on the basis of iridium(I)-diene chemistry in an aqueous medium (Scheme 6) [22]. 

**Scheme 7:**
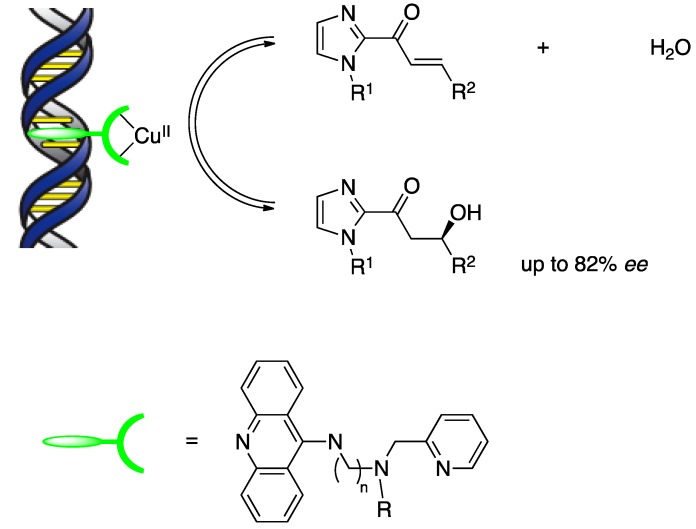
Catalytic enantioselective *syn* hydration of enones using DNA-based hybrid catalyst.

DNA-diene hybrid catalysts were prepared by introduction of bicyclo[2.2.2]octadiene ligands using a covalent anchoring strategy and examined in the iridium(I)-catalyzed allylic substitution of 1-phenyl acetate with morpholine. This work is significant in that the application of DNA-based asymmetric catalysis could be extended to organometallic chemistry as well as Lewis acid catalysis. In 2010, Feringa and Roelfes reported the first examples of a nonenzymatic catalytic enantioselective hydration of enones using a DNA hybrid catalyst and demonstrated the remarkable potential of this research field beyond traditional metal-ligand-based catalysis (Scheme 7) [27]. The hydration of α,β-unsaturated 2-acyl imidazole gave chiral β-hydroxyl ketone product with up to 82% *ee*. Unlike the above carbon–carbon bond-forming reactions in which second-generation ligands (e.g., dmbpy) were utilized successfully, the best results were obtained using first-generation ligands based on 9-aminoacridine.

## 4. Toward an In-Depth Understanding of DNA-Based Asymmetric Synthesis

### 4.1. Stereoinduction Mechanism

The use of DNA-based hybrid catalysts, as was stated above, led to high enantioselectivity, whereas the corresponding products were obtained as racemates in the absence of DNA. It is therefore clear that the enantioselectivity of the reactions originated from the DNA. However, the stereoinduction mechanism is not fully understood and remains a challenge. How does DNA induce and control enantioselectivity? To answer this question and elucidate the stereoinduction mechanism of DNA-based asymmetric catalysis, we should first clear up a problem about the DNA-binding mode of metal–ligand complexes (e.g., DNA groove binding *vs.* intercalation between base pairs). Besides groove binding and intercalation, there are several other modes for the reversible binding of small molecules with double-helical DNA such as electronic attractions of cations to the anionic phosphate backbone of DNA. However, in this review, we discuss mainly groove binding and intercalation, considering the structures of binding ligands.

**Scheme 8:**
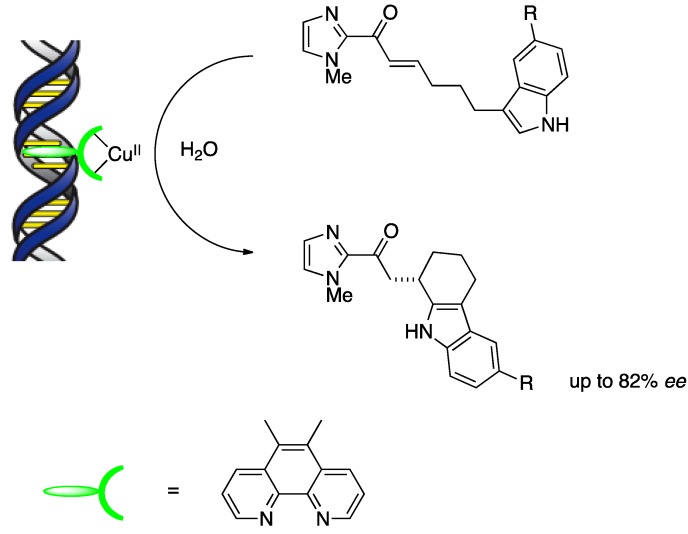
Asymmetric intramolecular Friedel–Crafts alkylations with a DNA-based hybrid catalyst.

Although a small molecule preferentially binds by either groove binding or by intercalation, depending on the structures of both the molecule and the DNA, there is also a possibility that one molecule employs more than one binding mode with DNA concurrently. It has therefore been a challenging task to evaluate the DNA-binding mode. Nevertheless, some experimental methods can distinguish between intercalation and groove binding, such as circular dichroism, linear dichroism, and X-ray crystallography. Viscosity and melting temperature analysis are also available for studying DNA-binding modes. For instance, intercalation induces unwinding and lengthening of the DNA and this DNA elongation gives rise to an increase in the relative viscosity. It is also well known that the melting temperature of duplex DNA stabilized by intercalators increases, while the melting temperature decreases for DNA bound by nonintercalators. With regard to the above DNA-based asymmetric catalysts, Feringa and Roelfes propose a mixture of binding modes including intercalation. Additionally, for Cu(dmbpy), they suggest that intercalation may not be the dominant binding mode because the melting temperature of DNA decreased slightly in the presence of Cu(dmbpy) [28]. On the other hand, Park and Sugiyama have recently developed asymmetric intramolecular Friedel–Crafts alkylations with a DNA-based hybrid catalyst and have suggested a plausible binding model based on intercalation (Scheme 8) [29]. Their suggestion is supported by experimental results that the melting temperature of the DNA oligomer increased slightly and the relative viscosity of DNA solution also increased in the presence of copper complexes.

In spite of the recent progress, further studies are necessary to determine DNA-binding modes. If we clearly elucidate the stereoinduction mechanism, including the binding mode of the metal complex to DNA, it will be a great step forward in DNA hybrid catalysis. It is expected that theoretical calculations and computer simulations based on molecular mechanics and density functional theory can be powerful tools to investigate the relationships between the helical chirality of DNA and the enantioselectivity of the chemical reaction. If we could predict metal–ligand complex–DNA interaction and design a new active site into a DNA scaffold using a theoretical approach, it would enable many interesting and useful applications such as rational tailoring of DNA-based hybrid catalysts.

### 4.2. Expanding the Synthetic Capabilities of DNA-Based Asymmetric Catalysis

**Scheme 9:**
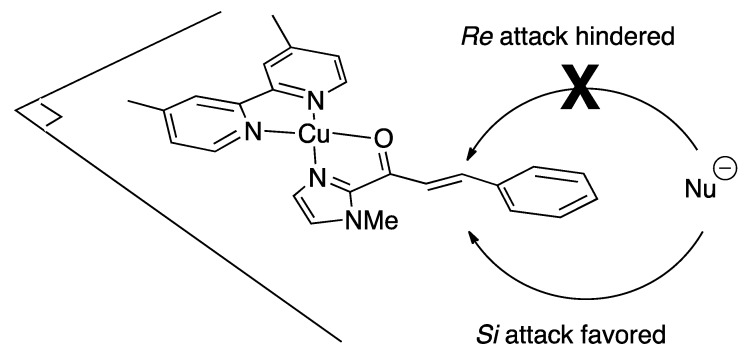
Stereofacial selectivity of the attack of the nucleophile in the DNA-based catalytic asymmetric Diels–Alder and Michael reaction of α,β-unsaturated 2-acyl imidazoles.

In DNA-based asymmetric catalysis, canonical right-handed B form DNA has generally been used. In the Diels–Alder reaction and Michael addition, the observed absolute configuration of the product can be explained as a consequence of the diene or nucleophile attacking the same *Si* face of the substrate because of the chiral environment provided by the DNA hybrid catalyst system (Scheme 9) [23,24,25]. 

In this case, how can we make the opposite enantiomer of the product? Theoretically, it is feasible if we use DNA hybrid catalysts based on L-DNA, which is the perfect mirror-image form of the naturally occurring D-conformation of DNA, a left-helical double helix. However, L-DNA is not a very attractive approach because of its impracticability compared with naturally occurring right-handed B-DNA. The other possible strategy is to use left-handed Z-DNA as a chiral scaffold. Recently, Sugiyama and co-workers have reported that Z-DNA could be used in intramolecular asymmetric Friedel–Crafts reactions [29]. In their work, contrary to the general expectation that left-handed Z-DNA would primarily induce the opposite enantiomer to that formed with right-handed B-DNA, Z-DNA gave only a decrease in enantioselectivity. Because, unlike L-DNA, Z-DNA has different physical properties in terms of solubility, duplex stability and selectivity with B-DNA, further study is needed on this issue.

**Scheme 10:**
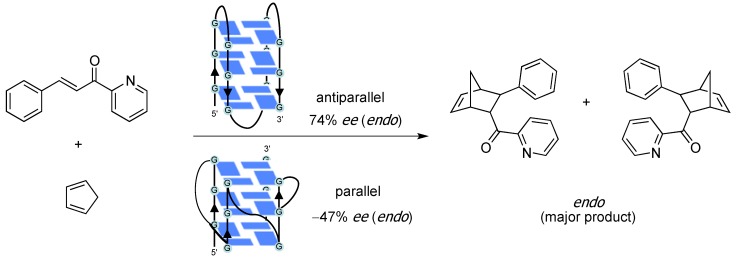
Diels–Alder reaction catalyzed by G-quadruplex DNA-based catalysts.

To return to the question above, the answer that enables DNA hybrid catalysts to control enantiomeric preference is a change of binding ligands. Recently, Roelfes and his group demonstrated that DNA hybrid catalysts based on the terpyridine-type ligands afforded the opposite enantiomer compared with the bipyridine ligands in copper(II)-catalyzed Diels–Alder and Friedel–Crafts reactions [30]. These results indicate that each enantiomer can be obtained selectively through the appropriate design of the ligand, even though the chirality of the DNA does not change. With regard to the structural diversity of the DNA, DNA can form not only a double helix, but also triplex and quadruplex forms. Very recently, G-quadruplex DNA-based catalysts have been developed and investigated based on the relationship between the changeable topology of the quadruplex structure and the catalytic reaction performance [31,32,33]. Li and co-workers demonstrated an enantioselective Diels-Alder reaction by using human telomeric G4DNA-based catalysts and reported the interesting results that the absolute configuration of the products could be reversed when the conformation of the G-quadruplex DNA was switched from antiparallel to parallel (Scheme 10) [32]. In DNA-based asymmetric synthesis, the result of a chemical reaction can change dynamically depending on the structure of the DNA and there are still a number of issues to be investigated.

## 5. Conclusions, Challenges and Perspectives

In conclusion, we have discussed the advent and the significant progress of DNA-based hybrid catalysts in asymmetric catalysis. DNA-based hybrid catalysts consist of DNA and a metal complex with a specific ligand through supramolecular or covalent anchoring strategies. They have been applied successfully in asymmetric carbon–carbon or carbon–heteroatom bond-forming reactions. However, challenges lie ahead. First, there are swings and roundabouts on the water solubility of DNA. Many organic reagents are not soluble in water, and some are unstable in water [34]. Can the DNA-hybrid catalytic system be applied in organic media? At this stage, it has been reported that up to 33% v/v of water-miscible organic solvents such as MeCN, DMF and alcohols can be applied without a decrease in enantioselectivity compared with DNA hybrid catalysis in water [35]. In addition, Liu’s group reported that DNA duplexes preformed in water are sufficiently soluble and stable in organic solvents in DNA-templated chemistry [36,37]. These hopeful results show that there is much room for improvement. Second, we should also consider the economic and practical perspectives. St-DNA is certainly a cheap biopolymer. However, if we use synthetic oligomers, overall costs must be further reduced for large-scale applications. When we consider the rapid development of science and technology, we can take an optimistic view with regard to this challenge. DNA hybrid catalysis will be more competitive with advances in DNA synthesis, including optimization of reaction conditions, improvement in coupling efficiency and the overall available quantities of the oligonucleotides. Although DNA hybrid catalysis is still in its infancy, the successful exploitation of helical chirality and significant progress in asymmetric catalysis suggest a bright future. We expect that an increasing number of scientists will explore this interesting and young research field and find broad application of DNA as a chiral scaffold for asymmetric syntheses.
